# GMDH-Guided Variable Prioritization in PAGE Block Growth of PEO-*b*-PAGE via Living Anionic Ring-Opening Polymerization

**DOI:** 10.3390/polym18111411

**Published:** 2026-06-05

**Authors:** Sangho Lee, Jong Dae Jang, Junhyung Bae, Tae-Hwan Kim

**Affiliations:** 1 Neutron Science Division, Korea Atomic Energy Research Institute, 111, Daedeok-daero 989 Beon-gil, Yuseong-gu, Daejeon 34057, Republic of Korea; shlee0724@kaeri.re.kr (S.L.); jdjang@kaeri.re.kr (J.D.J.); 2 Research Center for Advanced Nuclear Interdisciplinary Technology, Jeonbuk National University, Jeonju 54896, Jeonbuk State, Republic of Korea; 3 Department of Electrical Engineering, Daegu Catholic University, 13-13 Hayang-ro, Hayang-eup, Gyeongsan-si 38430, Gyeongbuk, Republic of Korea; 4 Department of Quantum System Engineering, Jeonbuk National University, Jeonju 54896, Jeonbuk State, Republic of Korea; 5 Division of Electronics and Information Engineering, Jeonbuk National University, Jeonju 54896, Jeonbuk State, Republic of Korea; 6 Department of JBNU-KIST Industry-Academia Convergence Research, Jeonbuk National University, Jeonju 54896, Jeonbuk State, Republic of Korea

**Keywords:** living anionic ring-opening polymerization, PEO-*b*-PAGE, temperature control, GMDH-guided analysis, GMDH modeling

## Abstract

The controlled synthesis of long hydrophobic blocks in amphiphilic block copolymers remains challenging in living anionic ring-opening polymerization (LAROP), particularly when competing effects such as back-biting and solubility limitations are involved. In this study, we investigated the temperature-dependent growth of poly(allyl glycidyl ether) (PAGE) blocks in PEO-*b*-PAGE block copolymers synthesized via LAROP using potassium naphthalenide as a co-initiator. Systematic variation in reaction parameters revealed that reaction temperature plays a significant role in governing effective PAGE block extension and dispersity control. Lower temperatures facilitated the formation of longer PAGE blocks with dispersities below 1.1 and *DP* values approaching targeted compositions, whereas elevated temperatures limited block growth. A group method of data handling (GMDH) polynomial neural network was employed as an auxiliary tool to prioritize influential variables within the experimental design matrix. The GMDH-guided analysis consistently identified temperature as the most influential variable, in agreement with experimental observations. These results provide quantitative insight into the temperature-controlled propagation behavior of PAGE in LAROP systems and offer a practical framework for improving block copolymer synthesis under kinetically and thermodynamically constrained conditions.

## 1. Introduction

Modern polymer research increasingly focuses on materials with controlled architectures, biocompatibility, and tunable physicochemical properties for applications including nanostructured materials and biomedical systems [1,2,3,4]. Among various polymer systems, block copolymers based on poly(ethylene oxide) (PEO) have attracted considerable attention because of their established biocompatibility and versatile functionality. In particular, amphiphilic block copolymers with narrow molecular weight distributions are advantageous for achieving reproducible material properties and predictable self-assembly behavior [1,2,5]. Living anionic polymerization provides an effective route for synthesizing such well-defined macromolecular architectures because chain growth proceeds from active chain ends while minimizing termination and chain-transfer reactions under carefully controlled conditions [6,7,8]. Ring-opening polymerization (ROP), particularly living anionic ring-opening polymerization (LAROP), further enables the controlled synthesis of polyether-based block copolymers through sequential monomer addition and regulated chain propagation [8,9].

Despite the controlled characteristics of LAROP under ideal conditions, the synthesis of block copolymers containing long hydrophobic poly(allyl glycidyl ether) (PAGE) segments remains experimentally challenging. When PAGE blocks are extended from hydrophilic PEO macroinitiators, the amphiphilic balance of the growing block copolymer progressively changes, which can reduce polymer solubility and promote aggregation or precipitation during polymerization [10]. In addition, temperature-dependent side reactions, including back-biting processes that may occur in flexible polyether-based systems, can further limit effective chain propagation [8,11]. As a result, achieving both low dispersity and substantial PAGE block extension remains difficult in practice. PEO-based poly(glycidyl ether) block copolymers, including PEO-*b*-PAGE systems, are nevertheless attractive because the allyl-functional side groups of PAGE enable versatile post-polymerization modification while maintaining the biocompatible characteristics associated with PEO [12,13,14,15,16]. Previous studies have reported PAGE homopolymers and PEO-*b*-PAGE block copolymers; however, the attainable PAGE molecular weights have often remained limited under conventional LAROP conditions [9,12,15].

Different hydrophobic block lengths can significantly influence the physicochemical behavior of amphiphilic block copolymers. Previous studies have shown that variations in block composition affect micelle size, stability, drug delivery system and self-assembly characteristics [17,18,19,20]. Therefore, precise control of hydrophobic block growth is important for tailoring the properties and potential applications of such materials. Consequently, the synthesis of well-defined block copolymers with precisely controlled block lengths remains a key objective in the development of advanced functional polymer systems.

Alongside experimental advances in polymer synthesis, data-driven analytical approaches have increasingly been explored to assist in reaction optimization, parameter screening, and interpretation of complex experimental datasets [21,22,23]. Machine learning-based methods can support the identification of correlations between synthesis parameters and experimentally observed material properties, thereby helping to reduce empirical trial-and-error experimentation. Among these approaches, the group method of data handling (GMDH) polynomial neural network provides a useful framework for prioritizing influential variables in nonlinear multivariate systems using relatively limited datasets [24,25]. However, such approaches should be interpreted as supportive analytical tools rather than replacements for mechanistic understanding or experimental validation, particularly in experimentally constrained polymerization systems.

In this study, we investigated the temperature-dependent growth behavior of hydrophobic PAGE blocks in PEO-*b*-PAGE synthesized via LAROP. A structured experimental design combined with GMDH-based variable analysis was employed to examine the relative influence of reaction parameters on PAGE block extension. The GMDH framework was used solely to assist in identifying influential synthesis variables and supporting experimental interpretation based on experimentally obtained datasets. The results demonstrate that reaction temperature exerts the strongest influence on achieving PAGE block lengths close to targeted compositions while maintaining narrow dispersity. These findings provide practical insight into the controlled synthesis of amphiphilic polyether block copolymers under kinetically and thermodynamically constrained conditions.

## 2. Materials and Methods

### 2.1. Materials

The following materials were used to prepare the block copolymers. PEO methyl ether (*m*PEO; *M*_n_ = 750 g/mol), an allyl glycidyl ether (AGE; Molecular weight = 114.144 g/mol) monomer, potassium metal (stored in mineral oil), naphthalene (99%), 2.0 M butyl magnesium chloride (in tetrahydrofuran; THF), 1.4 M sec-butyl lithium (in cyclohexane), dimethylformamide (99.8%, DMF), and anhydrous hexane (99%) were purchased from Sigma-Aldrich (St Louis, MO, USA). Tetrahydrofuran (99.8%, THF) was purchased from Junsei (Tokyo, Japan), and anhydrous methanol (99.9%) was purchased from Alfa Aesar (Ward Hill, MA, USA). Chloroform-*d* (99.8%, CDCl_3_) was purchased from Cambridge isotope (Tewksbury, MA, USA). THF and AGE were purified before use as described below.

### 2.2. Purification of AGE and THF

The AGE monomer was purified using 2.0 M butyl magnesium chloride for 30 min and degassed using three freeze–pump–thaw cycles in a vacuum (pressure < 10^−2^ torr) system. To eliminate the impurities in THF, 1.4 M *sec*-butyllithium was added, and the mixture was reacted for 30 min with vigorous stirring, followed by three freeze–pump–thaw cycles.

### 2.3. Synthesis of the Block Copolymers

The diblock copolymers of P(EO_17_-*b*-AGE_x_) were synthesized using LAROP. In this study, *m*PEO (*M*_n_ = 750 g/mol) was used as a fixed model macroinitiator rather than an optimized macroinitiator. The relatively short PEO block facilitated comparison of PAGE block growth under different reaction conditions and enabled straightforward monitoring of the PEO/PAGE *DP* ratio by ^1^H NMR at the laboratory scale. Because the LAROP process is extremely sensitive to trace impurities such as O_2_ and H_2_O, as well as other electrophilic contaminants (e.g., CO_2_), all experimental procedures were conducted under high vacuum (pressure < 10^−2^ torr). All glassware, including a 500 mL five-neck round-bottom reactor, was dried under vacuum at 60 °C overnight prior to use. Two amounts of *m*PEO (2 or 5 g) were used and dried in a vacuum reactor at 70 °C under continuous stirring. Subsequently, approximately 5 mL of *co*-initiator (0.4 M potassium naphthalenide solution) was injected into the vacuum reactor, followed by the direct injection of 10 mL of purified THF. After injecting the *co*-initiator, the *m*PEO solution turned dark green, and the initiation reaction was performed for 30 min. AGE monomers of different weights were added to each *m*PEO polymer solution, and the mixtures were propagated for experimentally predetermined reaction times of at least 14 h based on preliminary experiments and literature precedents for related LAROP systems. During polymerization, stabilization of the solution viscosity was qualitatively monitored as a practical indicator that chain propagation had substantially slowed. The reaction was subsequently terminated using anhydrous methanol. The final reaction solutions were precipitated into hexane to isolate the polymers. The precipitation was performed at a fixed solvent-to-hexane volume ratio of 10:90 (*v*/*v*), and identical mixing conditions were applied for all samples to minimize fractionation effects that could influence the apparent molecular weight distribution. The remaining hexane in the obtained block copolymer was evaporated under vacuum (pressure < 10^−2^ torr) for at least one day [9].

### 2.4. GMDH Polynomial Neural Network

The GMDH polynomial neural network is a useful algorithm for computer-based mathematical modeling and structure identification [24,25]. In GMDH, nonlinear model estimation equations are generated by hierarchically combining partial polynomial expressions based on quadratic equations of two variables. In each layer, regression analysis is performed within individual polynomial neurons generated from pairwise combinations of input variables. Node performance is evaluated using validation error, and only the best-performing neurons are retained for construction of the subsequent layer. Through this iterative process, the model progressively identifies the variables exerting the strongest influence on the target output parameter.

In this study, the experimentally designed reaction conditions listed in Table 1 were used as the input variables, while the experimentally obtained PAGE block growth behavior, represented by *DP*_PAGE_/*DP*_PAGE,calc_, was used as the target output parameter. The six input variables consisted of: (I) PEO molecular weight, (II) PEO mass, (III) co-initiator volume, (IV) AGE monomer content, (V) reaction time, and (VI) reaction temperature. The GMDH structure employed in this study consisted of three layers containing ten neurons each. The experimentally obtained dataset (*n* = 9) was analyzed using repeated random sub-sampling validation with 100 independent runs, in which the dataset was randomly divided into training and validation subsets during each run. Polynomial regression coefficients were fitted within each neuron, and validation performance was evaluated using the root-mean-square error (RMSE) and coefficient of determination (*R*^2^). No external datasets, synthetic data, or additional preprocessing procedures were used in this study.

### 2.5. Characterization of Block Copolymers

PEO-*b*-PAGE and PEO were analyzed by ^1^H NMR spectroscopy (500 MHz, INOVA 500, Palo Alto, CA, USA). All polymer samples were dissolved in CDCl_3_ at a concentration of 1 mg/mL. The number-average molecular weight (*M*_n_) of the PAGE block was estimated from ^1^H NMR integration using the known degree of polymerization of the PEO macroinitiator as a reference.

The molecular weight distribution and *Đ* of the polymers were evaluated by gel permeation chromatography (GPC; JASCO LC-4000, Tokyo, Japan). For GPC measurements, 5 mg of polymer was dissolved in 1 mL of DMF and stirred until a visually homogeneous solution was obtained prior to filtration through a 0.45 μm filter before injection. No obvious macroscopic insoluble residue was observed during sample preparation under the applied conditions, although the possibility of a minor insoluble fraction cannot be completely excluded for samples containing relatively long hydrophobic PAGE blocks. Accordingly, the reported GPC results primarily reflect the soluble fraction of the obtained block copolymers. The mobile phase was DMF at 40 °C, and calibration was performed using polystyrene standards. A refractive index (RI) detector was used for detection. Because conventional GPC provides apparent molecular weights based on hydrodynamic volume calibration, it was mainly used to compare molecular weight distributions and *Đ* trends among samples.

## 3. Results and Discussion

**Figure 1:**
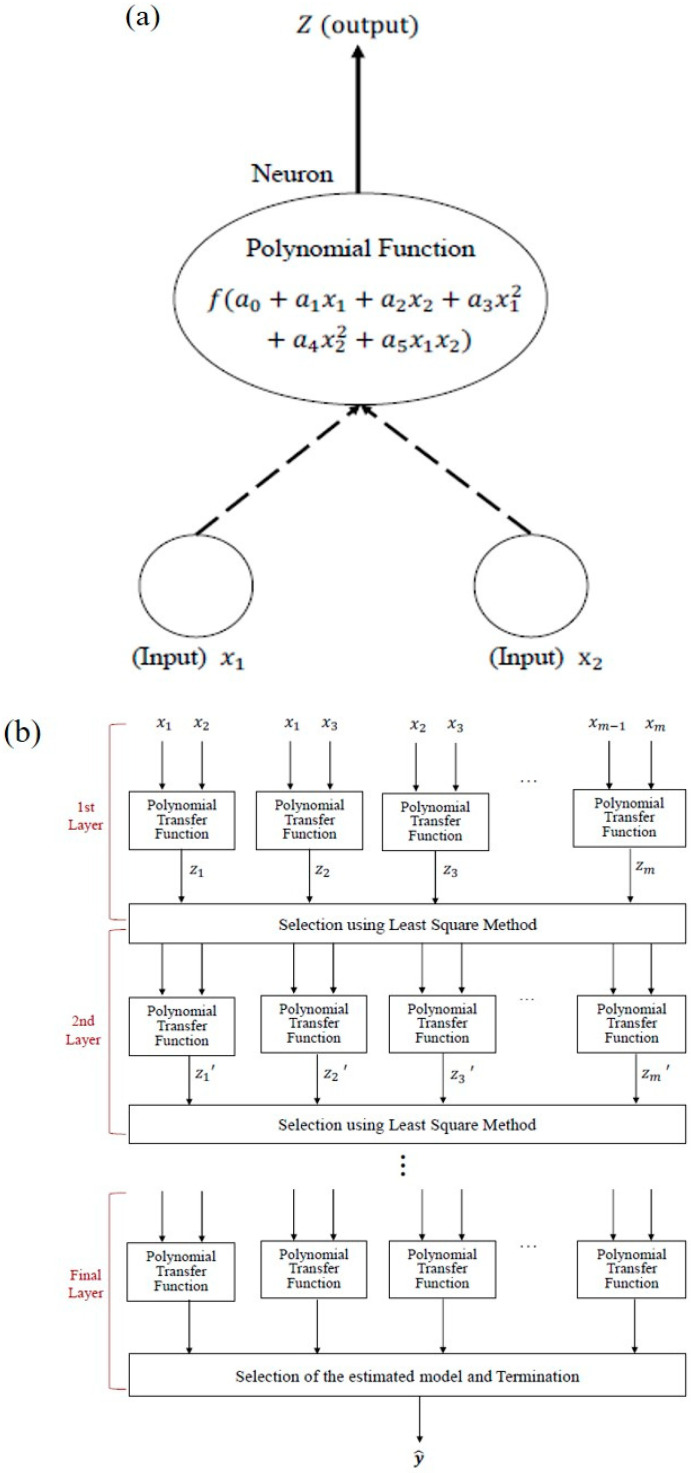
GMDH polynomial neural network design. (**a**) Basic structure of the polynomial neuron and (**b**) layers of the GMDH structure.

The experimentally obtained results, summarized in Table 1, exhibit clear variability in PAGE block growth depending on the applied reaction conditions. To systematically assess the relative influence of individual synthesis variables within this experimentally designed dataset, a group method of data handling (GMDH) polynomial neural network was applied as a post-analysis tool. The role of this analysis was to prioritize influential variables based on the available experimental data and to support the interpretation of the observed trends, rather than to predict polymerization outcomes beyond the measured dataset. The basic structure of a polynomial neuron used in the GMDH framework is shown in Figure 1a and is described by the following expression:

(1) $$z\left(x_{1},x_{2}\right)=a_{0}+a_{1}x_{1}+a_{2}x_{2}+a_{3}x_{1}^{2}+a_{4}x_{2}^{2}+a_{5}x_{1}x_{2}.$$

where *a*_*i*_ denotes the weight coefficients of the polynomial neuron and *x*_*i*_ represents the corresponding reaction variables. The multilayer structure of the GMDH model employed in this study is illustrated in Figure 1b. In the first layer, candidate input variables are combined pairwise and fitted using polynomial regression. Node performance is evaluated using a validation subset obtained by random sub-sampling, and only the best-performing nodes are retained for construction of the subsequent layer. This procedure is repeated until no further improvement in validation performance is achieved, resulting in a reduced subset of variables that exert the strongest influence on the formation of long hydrophobic PAGE blocks.

**Scheme 1:**
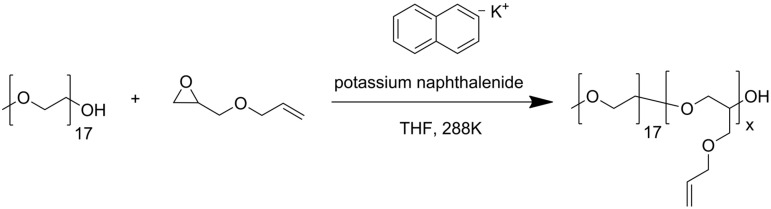
Schematic illustration of the LAROP of allyl glycidyl ether (AGE) initiated from a PEO macroinitiator activated by potassium naphthalenide (*co*-initiator), leading to the formation of PEO-*b*-PAGE block copolymers. The activation of the PEO chain end and subsequent block formation are illustrated.

Table 1 lists the initial experimental design for synthesizing PEO-*b*-PAGE block copolymers. The experimental conditions were designed to cover a range from small hydrophobic chain length ratios relative to PEO to large hydrophobic chain length ratios via LAROP. In the table, [*I*] is the initiator concentration, [*Q*] is the *co*-initiator concentration, and *DP* is the degree of polymerization. Here, the initiator concentration is defined as the concentration of the PEO macroinitiator that is activated by the *co*-initiator (potassium naphthalenide). The [*Q*]/[*I*] ratio is defined as the molar ratio of the *co*-initiator (potassium naphthalenide, [*Q*]) to the PEO macroinitiator ([*I*]), which determines the fraction of PEO chains that can be activated to initiate polymerization (Scheme 1).

The GMDH model was applied to analyze the experimentally designed dataset from the variables listed in Table 1 and identify the reaction parameters most strongly influencing PAGE block growth in PEO-*b*-PAGE block copolymers. The experimentally designed reaction conditions in Table 1 were used as the input variables, whereas the experimentally obtained PAGE block growth behavior, represented by the ratio *DP*_PAGE_/*DP*_PAGE,calc_, was used as the target output parameter for the analysis. In the model, the initial nodes corresponded to the six experimental variables from Table 1 (xi values): (I) PEO molecular weight (g/mol), (II) PEO mass (g), (III) *co*-initiator volume (mL), (IV) AGE monomer content (g), (V) reaction time (h), and (VI) reaction temperature (°C). These variables were combined pairwise within polynomial neurons and propagated through the multilayer GMDH structure shown in Figure 1b. The input dataset consisted of nine experimentally designed polymerization conditions (*n* = 9), each containing the six reaction variables described above. Model development was performed using repeated random sub-sampling validation with 100 independent runs. In each run, the dataset was randomly divided into training and validation subsets. Polynomial regression coefficients were fitted within each neuron, and node performance was evaluated based on validation error. Only the best-performing nodes were retained for construction of the subsequent layer. This iterative selection procedure was repeated until no additional improvement in validation performance was observed. No external datasets or synthetic data were used in this study.

**Figure 2:**
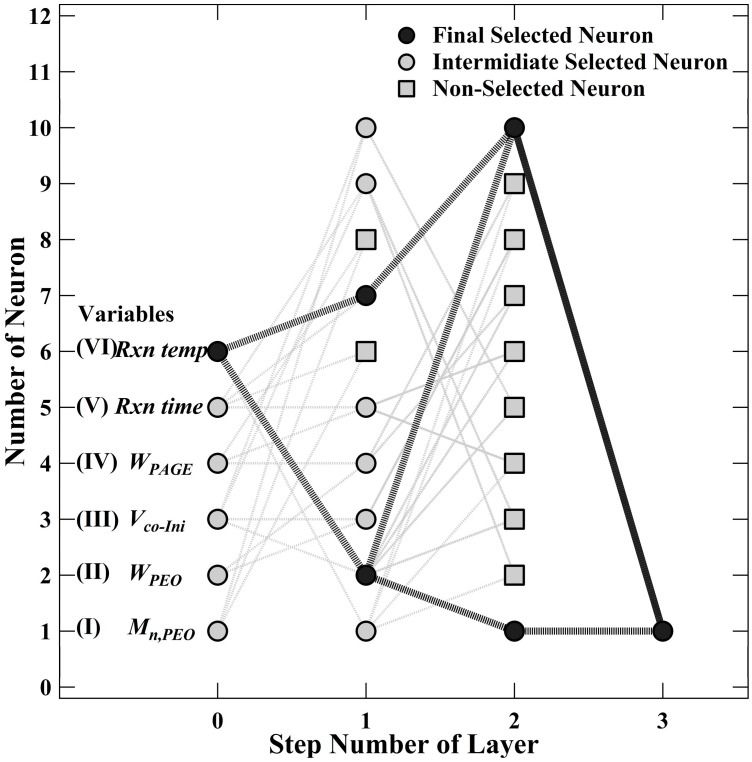
Results of GMDH polynomial neural network modeling. Black circle dots indicate selected neurons at each node, gray circle dots intermediate selected neurons at each node, whereas gray square dots indicate non-selected neurons. Black and gray lines represent network outputs originating from temperature that respectively reach and do not reach the final layer.

In this study, the GMDH polynomial neural network structure contained three layers comprising ten neurons each to identify the variables that affect the experimental results (Figure 2). The model was evaluated using repeated random sub-sampling validation (100 runs) to assess the robustness of variable selection and predictive performance. The first layer in Figure 2 (labeled 0) illustrates all six variables, which propagate through the layers of the model. At the end of the process, the reaction temperature (sixth node/variable) was the only remaining variable, indicating that it is the most influential variable for synthesizing block copolymers with long hydrophobic PAGE blocks. Each layer is the result of applying the $z\left(x_{1},x_{2}\right)$ matrix, and the red circle is a variable that shows a continuous effect in the repeated background experiment. By the third layer, reaction temperature remained the most consistently retained variable across repeated validation runs, whereas the other experimental variables exhibited comparatively weaker or less consistent contributions within the explored investigated conditions. Predictive performance was evaluated using the coefficient of determination (*R*^2^) and root-mean-square error (RMSE). Across repeated validation runs, the GMDH model exhibited stable performance, with an average RMSE of approximately 0.75 and an R^2^ value of about 0.92. Within this experimentally limited dataset (*n* = 9), reaction temperature consistently remained the most influential variable across network layers, indicating robustness of variable prioritization rather than independent predictive generalization.

To confirm the identification of reaction temperature as the most influential variable for forming long-chain PAGE blocks in PEO-*b*-PAGE block copolymer synthesis, we conducted LAROP experiments following the experimental design in Table 1. Molecular weight-related properties of the resulting block copolymers are listed in Table 2. *M*_n_,_NMR_ was determined by nuclear magnetic resonance (^1^H NMR) integration using the known *DP* of the PEO macroinitiator as a reference. *DP*_PAGE_ was calculated from the ratio of PAGE characteristic peaks to the PEO reference peaks. Selected polymerization conditions were repeated when sample availability allowed, and reproducibility was confirmed by repeated characterization. The uncertainty in *M*_n,PAGE_ calculated by ^1^H NMR arises primarily from peak integration and baseline correction, while *M*_w_ values from gel permeation chromatography (GPC) are affected by calibration with polystyrene standards and instrumental variability.

To interpret the experimental results, polymerization behavior was considered in light of prior studies on living ionic polymerization kinetics. Under ideal conditions, such systems often exhibit pseudo-first-order behavior, where ln([*M*]/[*M*_0_]) scales approximately with reaction time ([*M*] and [*M*_0_] denote the monomer concentration at a given time and the initial monomer concentration, respectively) [8]. This relationship suggests that the degree of polymerization can increase proportionally with monomer conversion in the absence of significant termination or side reactions. Consistent with this behavior, previous studies have reported well-defined PEO-*b*-PAGE block copolymers with controlled chain lengths obtained over reaction times of up to 24 h [12,13]. On the basis of previous reports for related LAROP systems and preliminary experimental observations, the polymerization reactions in the present study were propagated for experimentally predetermined periods of at least 14 h to promote sufficient PAGE block growth under the examined conditions. However, because real-time monomer conversion measurements were not continuously performed during polymerization, the applied reaction times should be interpreted as experimentally selected propagation periods rather than rigorously determined complete-conversion endpoints. Deviations from the targeted degree of polymerization are therefore interpreted in terms of practical limitations, such as temperature-dependent side reactions and solubility effects, rather than incomplete initiation.

**Figure 3:**
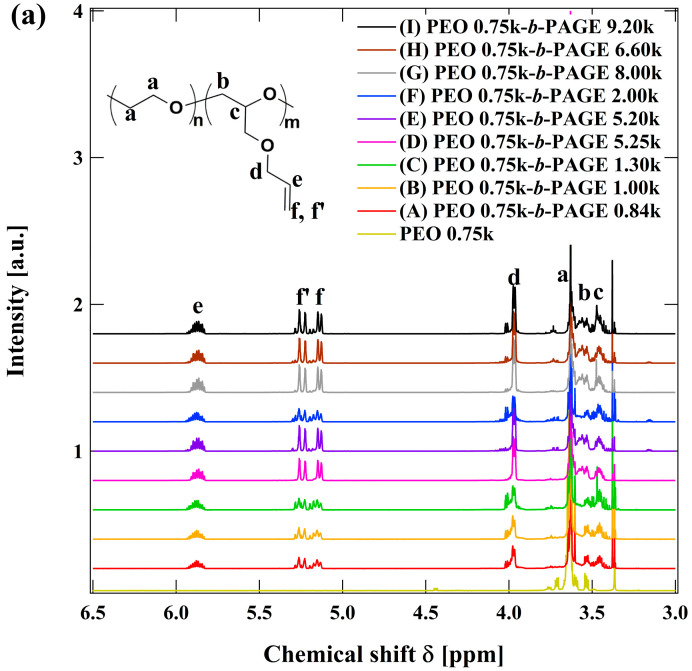

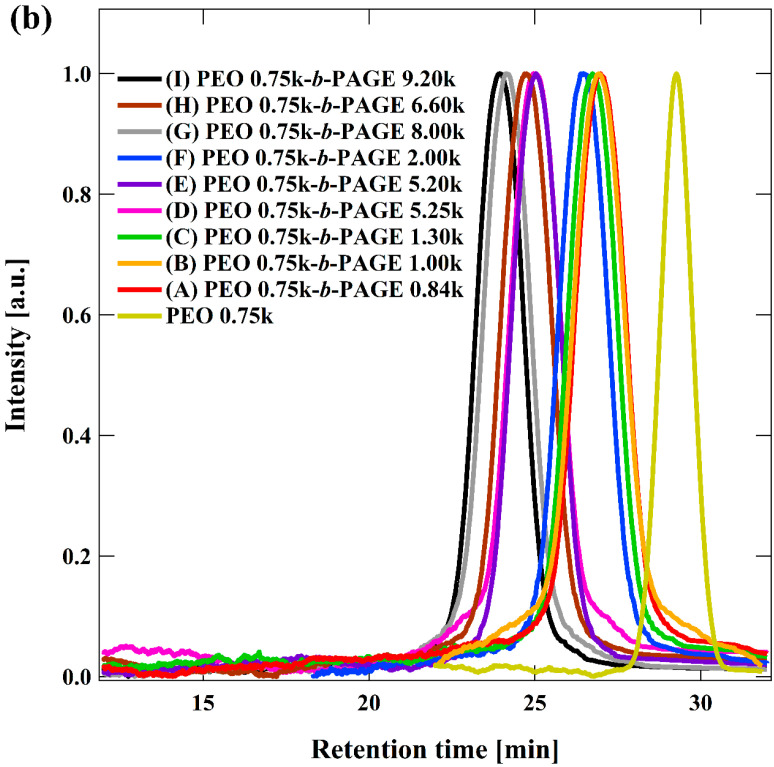
(**a**) ^1^H NMR spectra (3.0–6.5 ppm) of the commercially available PEO macroinitiator and the obtained PEO-*b*-PAGE block copolymers. All block copolymer samples contain the same PEO block, and the PAGE block length was estimated from the integral ratio between the characteristic PAGE signals and the PEO ether proton (**a**,**b**) reference region, which partially overlaps with neighboring PAGE-related polyether signals in the block copolymer spectra. Chemical shifts were referenced to residual chloroform (CDCl_3_, 7.2 ppm). (**b**) GPC traces of the corresponding PEO-*b*-PAGE samples, used to compare the *Đ* among different polymerization conditions.

The synthesis results for the various monomer concentrations were characterized using ^1^H NMR spectroscopy and GPC. As shown in Figure 3a, ^1^H NMR was used to estimate the number-average molecular weight of the PAGE block (*M*_n_,_NMR_). A commercially available PEO macroinitiator with a specified molecular weight (*M*_n_,_PEO_) was used as the starting material. Specifically, *M*_n_,_NMR_ was determined from ^1^H NMR integration using the known degree of polymerization (*DP*) of the PEO macroinitiator (Table 1) as a reference, and *DP*_PAGE_ was calculated from the integration ratio of PAGE characteristic proton signals to the PEO reference signals. Figure 3b shows the GPC traces of the obtained block copolymers, which were used to evaluate *Đ* of the samples. In this work, *M*ₙ was primarily estimated by ^1^H NMR because conventional GPC based on hydrodynamic volume may lead to systematic deviations for amphiphilic block copolymers such as PEO-*b*-PAGE. The resulting *Đ* values were below 1.1 for all samples, indicating narrow molecular weight distributions.

Comparing the targeted and experimentally obtained degrees of polymerization of the PAGE block (*DP*_PAGE_) (Table 1 and Table 2, respectively) shows that conditions (A), (B), (E), and (G), with *DP*_PAGE_ above 122, resulted in substantially lower experimental *DP*_PAGE_ values. This is explained by the relationship between concentration, reaction rate, molecular weight of the polymers, and solubility. Generally, the kinetics of living anionic polymerization are as follows:

−d[*M*]/d*t* = *k*_p_[*P*^−^][*M*] = *k*_p_[*I*][*M*] (2)

where *k*_p_ is the propagation reaction rate constant, [*P*^−^] is the activated anionic site concentration during propagation, and *t* is the reaction time. In this study, the reaction time (or the resulting chain length) did not show a clear dependence on [*Q*], which was varied from 0.18 to 1.5 ([*Q*] = [*KO*]/[*OH*], where [*KO*] is the molar amount of K-naphthalenide and [*OH*] is the molar amount of hydroxyl groups in PEO. This showed that the *co*-initiator was activated enough, and the additional *co*-initiator did not affect the reaction rate. Increasing [*M*] increases *k*_p_, while decreasing [*I*] increases the *M_w_* of the polymer, which decreases the solubility of the polymer enthalpically according to the Flory–Huggins regular solution theory. As a result, the polymer precipitates out of the solvent under these conditions, and the reaction stops. Sudo demonstrated that ionic polymerization can be terminated via back-biting reactions after sufficient polymer-chain propagation when the chemical structure of the backbone chain is flexible [11]. In addition, back-biting reactions are enhanced at higher reaction temperatures.

**Figure 4:**
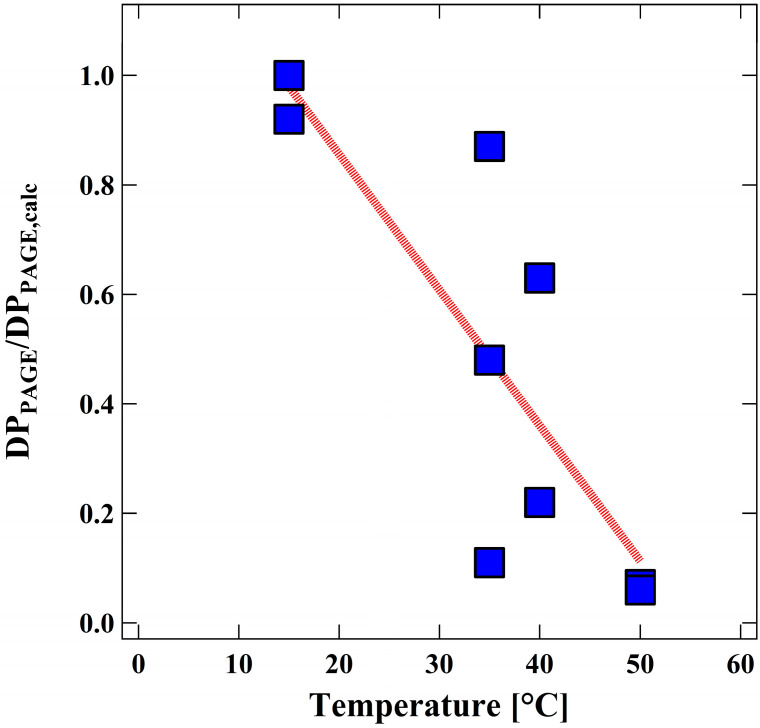
Ratio of the experimental to target degree of polymerization of the PAGE block (*DP*_PAGE_/*DP*_PAGE,calc_) as a function of reaction temperature for PEO-*b*-PAGE block copolymer synthesis. Each data point corresponds to a polymerization condition listed in Table 2. The plot highlights the strong temperature dependence of PAGE block growth, suggesting that lower reaction temperatures are associated with PAGE block growth closer to the targeted degree of polymerization within the examined experimental investigated conditions.

The GMDH-based analysis of the experimentally obtained dataset indicated that temperature was the most influential reaction variable. To verify this, PEO-*b*-PAGE was synthesized at various temperatures, monomer concentrations, initiator concentrations, and reaction times. To better visualize the deviation from the targeted PAGE block length, *DP*_PAGE_/*DP*_PAGE,calc_ is plotted as a function of reaction temperature (Figure 4).

Conditions (A), (B), (C), and (D) (Table 2), all conducted at reaction temperatures of 40–50 °C, resulted in *DP*_PAGE_/*DP*_PAGE,calc_ values of 0.07–0.63, respectively. In contrast, conditions (E), (F), and (G), performed at 35 °C, exhibited comparatively higher *DP*_PAGE_/*DP*_PAGE,calc_ values of 0.11, 0.87, and 0.48. Polymerizations carried out at 15 °C produced *DP*_PAGE_/*DP*_PAGE,calc_ values approaching unity. These results indicate that lower reaction temperatures favor more effective PAGE block growth relative to the targeted compositions. The improved agreement between targeted and experimental PAGE block lengths at reduced temperatures suggests suppression of side reactions that may limit effective chain propagation at elevated temperatures. Other experimental variables were also varied within the dataset. Temperature showed the strongest association with PAGE block growth within the explored investigated conditions. However, because the present experimental design involved simultaneous variation in multiple parameters, these observations should be interpreted qualitatively rather than as isolated quantitative comparisons. Previous work on PAGE homopolymer synthesis reported that protic impurities introduced during initiator preparation can interfere with polymerization at higher temperatures [12]. In addition, temperature-dependent side reactions, including back-biting processes in flexible polyether backbones, have been discussed in the context of ionic polymerization [11]. The propagation step in anionic polymerization involves ion pairs between the growing anionic chain end and the counter-ion. The distribution among tight, loose, and free ion pairs depends on reaction conditions, including temperature, solvent, and initiator system. Although the exact fractions are system-specific, temperature-dependent shifts in ion-pair equilibria can influence effective propagation rates. Under the present conditions, reduced reaction temperatures may favor propagation relative to competing side reactions, thereby enabling greater PAGE block extension. Taken together, the experimental observations demonstrate a pronounced temperature dependence in the LAROP growth of PAGE blocks. Within the multivariable experimental investigated conditions examined in this study, reaction temperature showed the strongest apparent correlation with achieving PAGE block lengths close to the targeted values. However, because multiple reaction parameters were varied simultaneously, the present results should not be interpreted as a strictly isolated single-variable temperature study.

Application-specific characterization was beyond the scope of the present study. However, the practical significance of controlling PAGE block growth extends beyond the polymerization process itself. Amphiphilic PEO-*b*-PAGE block copolymers are recognized as versatile materials for self-assembled nanostructures because the balance between the hydrophilic PEO segment and the hydrophobic PAGE segment strongly influences micelle formation, aggregation behavior, and nanoscale morphology [1,2,5,10,25]. Precise control of PAGE block length therefore provides a direct means of tuning self-assembly characteristics, including particle size, core–shell structure, and colloidal stability in aqueous environments. Such control is particularly important for the rational design of nanocarriers and delivery systems, where structural uniformity and reproducible assembly behavior are critical for achieving predictable performance. In addition, the allyl functionalities present along the PAGE block provide reactive sites for a wide range of post-polymerization modification reactions, including thiol–ene click chemistry and other orthogonal conjugation strategies [9,12,13]. This chemical versatility enables the introduction of bioactive molecules, fluorescent probes, targeting ligands, ionic groups, or crosslinkable moieties without altering the underlying polymer backbone. Consequently, PEO-*b*-PAGE copolymers serve as attractive platform materials for the preparation of functional nanomaterials, surface-modified nanoparticles, and responsive polymer systems [26,27,28].

Furthermore, control over PAGE block length may influence phase behavior, chain packing, and intermolecular interactions in concentrated systems. These characteristics are relevant to the development of soft materials such as physically or chemically crosslinked hydrogels, stimuli-responsive networks, and nanostructured polymer assemblies [2,3,4,15]. Because the hydrophilic–hydrophobic balance can be systematically adjusted through PAGE block extension, the synthetic strategy presented in this study provides a practical route toward tailoring physicochemical properties for future applications in drug delivery, nanocarriers, self-assembled materials, and advanced functional polymer platforms [28,29]. Although application-oriented characterization was beyond the scope of the present work, the ability to reproducibly synthesize PEO-*b*-PAGE block copolymers with controlled PAGE block lengths represents an important prerequisite for these future developments.

While these potential applications highlight the broader significance of PAGE block length control, the present study focused primarily on identifying the synthesis variables governing PAGE block extension under LAROP conditions. The observed sensitivity hierarchy should therefore be interpreted within the investigated reaction space and may differ for other monomer, initiator, or solvent systems. Consequently, the GMDH-based variable tracking framework should be retrained or re-tuned when applied to different polymerization systems, particularly when only limited experimental datasets are available. Alternative explainability approaches, such as Shapley additive explanation (SHAP)-based analysis [30], may provide complementary insight into variable importance and nonlinear parameter interactions in future studies as larger experimental datasets become available.

## 4. Conclusions

In this study, we examined the temperature-dependent growth behavior of hydrophobic PAGE blocks in PEO-*b*-PAGE synthesized via living anionic ring-opening polymerization (LAROP). Systematic variation in reaction parameters demonstrated that reaction temperature strongly influences the achievable PAGE block length relative to targeted compositions. Lower temperatures enabled PAGE block growth closer to the calculated degree of polymerization while maintaining narrow dispersity (*Đ* < 1.1), whereas elevated temperatures limited effective chain extension. The observed temperature sensitivity is consistent with practical limitations associated with temperature-dependent side reactions, ion-pair equilibria, and solubility constraints in anionic polymerization systems. These findings provide mechanistic insight into the constraints governing hydrophobic block extension in PEO-*b*-PAGE and highlight the importance of precise temperature control when targeting high block asymmetry.

A GMDH-based analytical approach was employed as a supplementary tool to prioritize influential variables within the experimentally designed dataset. The analysis consistently identified temperature as the most influential variable, in agreement with experimental observations. Overall, this combined experimental and data-guided framework offers a practical strategy for reducing empirical iteration in complex LAROP systems while maintaining mechanistic interpretability. Because the relative hydrophilic–hydrophobic balance of the block copolymers can be adjusted through PAGE block extension, the obtained polymers may provide a useful platform for future studies investigating tunable self-assembly behavior, phase separation, and other application-related physicochemical properties. Future studies may further investigate how variations in PAGE block length influence thermal transitions, crystallization behavior, and phase separation characteristics using complementary thermal and structural characterization techniques. Although industrial-scale implementation of LAROP remains challenging because of its sensitivity to impurities, the method remains valuable for the synthesis of well-defined block copolymers used in self-assembled and nanostructured materials. Beyond providing mechanistic insight into temperature-controlled PAGE block growth, the present findings establish a practical foundation for the future design of PEO-*b*-PAGE block copolymers with tailored self-assembly behavior and physicochemical properties, thereby supporting their potential application in nanocarriers, responsive polymer systems, functional polymer platforms, and hydrogel-based materials.

## Figures and Tables

**Table 1:** Reaction conditions used as input variables for the GMDH polynomial neural network analysis and the corresponding LAROP experiments to synthesize PEO-*b*-PAGE block copolymers. Conditions (A) and (B) were repeated runs under identical conditions to confirm reproducibility.

Sample Code	*M*_n,PEO_ (g/mol)	*W*_PEO_ ^a^ (g)	*V_co_*_-Initiator_ ^b^ (mL)	[*Q*]/[*I*] ^c^ (–)	*W*_AGE_ ^a^ (g)	Reaction Time (h)	Reaction Temp. (°C)	Target *DP*_PAGE_ (–)
(A)	750	5	3	0.18	20	20	50	122
(B)	750	5	3	0.18	20	20	50	122
(C)	750	5	7	0.3	20	14	40	51
(D)	750	5	7	0.3	20	20	40	73
(E)	750	2	1	0.15	32	14	35	410
(F)	750	2	8.5	1.275	8	24	35	21
(G)	750	5	3	0.18	20	24	35	146
(H)	750	2	10	1.5	32	16	15	58
(I)	750	2	8	1.2	32	16	15	88

^a^ *W*_PEO_ and *W*_AGE_ denote the masses of PEO methyl ether (macroinitiator) and AGE monomer, respectively. ^b^ *V*_initiator_ denotes the volume of the initiator solution added. ^c^ [*I*] is the initiator concentration of the PEO macroinitiator, and [*Q*] is the *co*-initiator concentration (K-naphthalenide); thus, [*Q*]/[*I*] is a unitless molar ratio (−; unitless expression).

**Table 2:** Molecular characteristics of the synthesized PEO-*b*-PAGE block copolymers obtained under the experimental conditions described in Table 1.

Sample Code	*M*_n,PEO_ ^a^ (Specified) (g/mol)	*DP*_PEO_ ^b^ (–)	*M*_n_,_PAGE_ ^c^ (NMR) (g/mol)	*DP*_PAGE_ ^b^ (–)	Block Mass Ratio (*W*_PAGE_/*W*_PEO_) ^b^ (–)	*DP*_PAGE_/*DP*_calc_,_PAGE_ ^b^ (–)
(A)	750	17	1000	9	1.33	0.07
(B)	750	17	840	7	1.12	0.06
(C)	750	17	1300	11	1.73	0.22
(D)	750	17	5250	46	7.00	0.63
(E)	750	17	5200	46	6.93	0.11
(F)	750	17	2000	18	2.67	0.87
(G)	750	17	9200	70	10.67	0.48
(H)	750	17	6600	58	8.80	1.00
(I)	750	17	8000	81	12.27	0.92

^a^ *M*_n,PEO_ (specified) is the supplier-provided number-average molecular weight of the PEO methyl ether macroinitiator, ^b^ *DP* and ratio terms are unitless (−; unitless expression), and the block mass ratio is reported as *W*_PAGE_/*W*_PEO_. ^c^ *M*_n,PAGE_ (NMR) was estimated from ^1^H NMR peak integration.

## Data Availability

All data are available in the main text.
